# Tenure Track Policy Increases Representation of Women in Senior Academic Positions, but Is Insufficient to Achieve Gender Balance

**DOI:** 10.1371/journal.pone.0163376

**Published:** 2016-09-29

**Authors:** Martha M. Bakker, Maarten H. Jacobs

**Affiliations:** Department of Environmental Sciences, Wageningen University, Wageningen, The Netherlands; Iowa State University, UNITED STATES

## Abstract

Underrepresentation of women in senior positions is a persistent problem in universities worldwide, and a wide range of strategies to combat this situation is currently being contemplated. One such strategy is the introduction of a tenure track system, in which decisions to promote scientific staff to higher ranks are guided by a set of explicit and transparent criteria, as opposed to earlier situations in which decisions were based on presumably more subjective impressions by superiors. We examined the effect of the introduction of a tenure track system at Wageningen University (The Netherlands) on male and female promotion rates. We found that chances on being promoted to higher levels were already fairly equal between men and women before the tenure track system was introduced, and improved–more for women than for men–after the introduction of the tenure track system. These results may partly be explained by affirmative actions, but also by the fact that legacy effects of historical discrimination have led to a more competitive female population of scientists. In spite of these outcomes, extrapolations of current promotion rates up to 2025 demonstrate that the equal or even higher female promotion rates do not lead to substantial improvement of the gender balance at higher levels (i.e., associate professor and higher). Since promotion rates are small compared to the total amount of staff, the current distribution of men and women will, especially at higher levels, exhibit a considerable degree of inertia—unless additional affirmative action is taken.

## Introduction

Gender bias in academia, favouring men over women, penetrates virtually all domains of academia. Consequential inequality includes positions [1–4], promotions to higher positions [5], income levels at equal positions [6,7], success in obtaining grants [8,9], authorship of peer reviewed papers [10–14], quality evaluations of track records [15], and student evaluations [16] (but see [17] for different findings). Naturally, not all conclusions drawn in specific studies are univocally accepted. As an example, the question whether figures on grant assignment by the Dutch Science Organization suggest gender bias is heavily debated [18–20]. Also, the existence of gender disparity in some specific domains of academic work is questioned. For example, while one meta-analysis suggests gender bias in peer review of grant proposals [21], subsequent meta-analyses using multilevel analyses that allow nested data, favoured the null-hypothesis of gender similarity [22,23] (note that these studies address peer review only, and hence do not preclude the existence of gender bias in grant awarding). Yet, if all figures indicating gender bias in academia would be artefacts, for instance due to sampling error (i.e., by coincidence or on purpose, a-typical cases were selected hampering the generalizability of findings) or flaws in statistical analyses, one would expect equal amounts of studies suggesting bias favouring men and bias favouring women. The latter studies do exist in the literature [24], but are by far outnumbered by the former. Hence, convergent evidence is so evocative that denying gender bias in academia would be equivalent to denying climate change.

From a normative point of view, gender bias is problematic as it flags unequal treatment of women and men in academia, under the assumption that there is no principal difference in capacity potential between sexes. Equal rights to women and men are expressed in the constitutions of many nations, as well as in the UN human rights resolution. Underrepresentation of women in academia seems at odds with this ideology. Gender inequity is also problematic in the light of efficiency in academia, if underrepresentation of a large pool of equally capable workforce leads to a suboptimal overall quality level of work.

By and large, existing research has focused on demonstrating gender bias, and exploring the various domains into which the bias extends. Such research is valuable as it demonstrates the existence of gender inequity and hence points to a societal problem. Also, knowledge on gender imbalances in different areas is important because potential measures to mitigate bias would be very different across these areas. For example, actions to reduce differences in income levels between women and men at equal positions could include raising awareness and producing guidelines for managers, and raising awareness amongst women so they can improve negotiation positions. Actions to reduce acceptance rates of peer-reviewed articles could include establishing rigorous double-blind review systems that rule out any suggestion about the sex of the author(s). Ultimately, reducing gender bias requires effective interventions in current practices, as gender bias is persistent and unlikely to fade away without deliberate action.

This article makes a novel contribution to the literature by investigating the effect of an intervention on gender bias in promotion to higher academic positions. Specifically, we examined the effect of the introduction of a tenure track system (TTS) in 2010 at Wageningen University, The Netherlands, on gender bias in promotions to higher positions, and estimated the potential impact on gender distributions in the near future.

## Theoretical Framework

Probably, gender bias in academia is, in many nations, not a feature of deliberate and explicit will to suppress women. Rather, underrepresentation of women is often assumed to result from subconsciously operating biases that influence decisions [9,25,26]. In this respect, psychologists have made a relevant distinction between two modes of thinking and decision making, often labelled system 1 and system 2 [27]. System 1 is operating “automatically and quickly, with little or no effort and no sense of control”; while system 2, on the other hand, “allocates attention to the effortful mental activities that demand it” [27]. System 1 is associated with automatic and therefore implicit thinking and judging, while system 2 is associated with explicit reasoning that requires continuous attention. As explicit reasoning is effortful and our mental capacities for doing so are limited and easily depleted, we usually rely on system 1. System 1 also perpetually feeds system 2 with impressions and suggestions, and system 2 often takes these over as a basis to take explicit decisions.

System 1 has innate tendencies, yet is plastic and open to experiential learning, such as implicit learning about subtle social rules that guide the co-ordination and behaviour amongst members of a cultural community. System 1 is not only fed by first-hand experiences, but by mediated experiences as well–images and stories propagated by social agents (peers, media, family, education). Many of these messages advertise–more or less subtle–differences between the sexes and assign different roles, behaviours, and capacities to the sexes, thus producing gender as stereotyped implicit cognitions. These stereotyped cognitions are likely to be employed in system 1 thinking, in a non-explicit and subconscious, and therefore unreflective mode. As system 1 thinking is prone to bias [27], these stereotypes might play a role as prejudice towards women and men in guiding decision-making. Through this mechanism, the cognitive heuristics of system 1 may produce gender bias in decision-making relevant to academic careers.

Organizational structures can influence thinking of affiliated individuals [28] and thereby affect the balance between system 1 and 2 usage in particular domains of thought. An important manner of influencing this balance is through introducing explicit criteria for considering actions and decisions. Explicit criteria are likely to evoke system 2 thinking in individuals, and hence leave less room for unreflective system 1 direction of action and decision-making. Thus, if promotions to higher positions depend largely on the decision of the direct superior (as was the case at Wageningen University before the introduction of the tenure track system, see also the Context section), and less on explicit and transparent criteria, these decisions are relatively more likely to be fed by system 1, and hence prone to gender bias subconsciously influencing these decisions. Introduction of a tenure track system (TTS) fosters decisions about promotion by committees and by and large on the basis of explicit and transparent criteria. It is more likely that these decisions are fed by explicit system 2 reasoning, and hence less prone to automated stereotype thinking. We do not claim that a one-to-one relationship exists in the sense that promotion decisions before tenure track policy would be exclusive system 1 decisions, and after the policy system 2 decisions. Rather, we claim a relatively larger role of system 1 in the former situation, and a relatively larger role of system 2 in the latter. Under tenure track policy, then, chances of women to be promoted to higher positions could increase. Therefore, we hypothesize that the introduction of a tenure track system improves the representation of women in higher academic positions at Wageningen University. Previous research suggests processes of evaluation to play a key role in the advancement of women in science [29].

However, as several scientific studies suggest that the presumably objective criteria in the TTS, such as number of publications, student evaluations, and successful grant applications are in itself prone to gender bias (see Introduction), the TTS could be just another mechanism that reinforces the status quo of underrepresentation of women. Moreover, within the TTS, judgement of academic quality is still to some extent based on subjective appreciation by members of the evaluation committee, and it remains to be seen if this subjective element–particularly in interaction with the potential negative bias in quantitative scores–will not continue to lead to underrepresentation of female scientists in senior academic positions [30,31]. For these reasons, our hypothesis is not self-evident and merits testing. In this article, we will present figures reflecting gender distributions over the years 2006 to 2014, and test the hypothesis that TTS increases promotion rates of women. In addition, we will extrapolate observed promotion rates to estimate gender distributions for 2025 under TTS policy, in order to evaluate the effectiveness of the TTS for achieving gender balance.

## Methods

### Context

Wageningen University is a relatively small university (approximately 10,000 students and 6,500 staff distributed over the university and the associated research institutes) in The Netherlands that used to be focused on the agricultural sciences. Over the past decades, the university has widened the scope to include food and food production, the living environment, and health, lifestyle and livelihood. It is currently divided into the following five departments: Environmental Sciences, Plant Sciences, Animal Sciences, Social Sciences, and Agrotechnology & Food Sciences. In spite of a relatively egalitarian culture of The Netherlands [32], women are drastically underrepresented in university staff at Dutch universities [33]. In addition, women are more underrepresented in higher positions (e.g., 17.1% of full professors is female in 2015). Underrepresentation at Wageningen University is larger than the average underrepresentation at Dutch Universities (e.g., 7.6% of full professors is female).

Promotions to higher ranks traditionally (i.e. before the introduction of the TTS) happened as follows: promotions from junior to senior assistant professor, or from junior to senior associate professor typically occurred when a scientist reached the end of the salary scale for that position, and when his or her performance was on average more than satisfactory—a judgement made by the chair holder. Chair holders, always full professors, lead the research groups within Wageningen University, and as such have authority to take important decisions for that group. Before TTS introduction, promotions from assistant to associate, or from associate to full professor only occurred whenever a position (for associate or full professor) became available. In case there was more than one candidate interested in that position, the chair holder would take the decision of who to appoint. Appointments of new staff (mostly as assistant professor) were also done by the chair holder, based on his or her impression of the quality of the candidate.

TTS was introduced at Wageningen University in 2010, roughly around the same time many other Dutch Universities started with a TTS. TTS has been introduced to improve the quality of scientific staff, as it was expected to: (1) attract young talent, by offering them a position with a better future perspective than a postdoc positon; (2) facilitate and stimulate the development of scientific talent, irrespective of whether positions for associate or full professors are available or not; and (3) improve the objectiveness of the evaluation of the scientists, leading to better perspectives of current minorities in higher scientific ranks (e.g. women, non-western scientists) [34]. Although the Dutch TTS is inspired by the TTS as implemented at U.S. universities, it differs in that it needs to account for the maximum duration of fixed-term contracts, which is six years in The Netherlands. This means that only the first part of the track (i.e. from assistant professor to associate professor) is done in a fixed-term position, while the second part (i.e. from associate professor to full professor) is done when the scientist is already tenured. In The Netherlands, and thus in this paper, the term tenure track stands for the entire track of promotions from junior assistant professor to full professor. As such, the TTS encompasses more than the set of procedures that lead to a tenured position; it stands for the entire set of procedures that define the rules for promotions to higher ranks, for both tenured and non-tenured scientific staff.

The procedures for a promotion under the TTS at Wageningen University are as follows: a candidate requests a promotion and submits an overview of obtained credits. Credits can be obtained by publishing papers, acquiring research grants, supervising PhD students, and teaching–provided that student evaluations are favourable. A secretary from the human resources department checks whether the obtained credits comply with the minimum requirements for the desired promotion. If so, a promotion advisory committee is established, for which the candidate prepares a portfolio that includes, next to an explication of the obtained credits, a research vision and plan. Based on this portfolio and an interview with the promotion candidate, the committee decides whether the request for promotion is granted. Thus, under TTS policy, decisions about promotions were transferred from the chair holder to the advisory committee. For appointments to assistant professor (i.e. entries into the tenure track), only a judgement of publication record and H-index suffices, but still a committee is appointed to make this judgement.

### Data

At the Human Resource Management (HRM) department of Wageningen University, a database is kept that lists all scientific staff and their ranks, which is updated at yearly basis. For the purpose of this study, an extract of this database for the years 2006 to 2014 was made available to us by the HRM department, in which all personal identifying information was deleted and only gender and rank information was kept. The period 2006 to 2014 was selected as TTS was introduced halfway 2010, so it contains an equal amount of years before and after TTS introduction. Within Wageningen University, the following ranks exist: junior assistant professor (UD2), senior assistant professor (UD1), junior associate professor (UHD2), senior associate professor (UHD1), and professor (PH). These ranks are the same at all Dutch Universities, and are comparable to the U.S. ranks of assistant, associate, and full professor and to the British ranks of lecturer (assistant professor), senior lecturer (associate professor), and professor. The difference between junior and senior (both within assistant and associate professorship) lies in requirements for publication rates, student evaluations, managerial responsibilities, and number of PhD promotions. An ‘entry’ is defined as an appointment to UD2 (from outside the university or from the pool of junior researchers and PhD students within the university), while a promotion is defined as any change in rank towards a higher one. It was assumed that any entry or promotion between 2006 and 2010 happened according to the traditional procedure (decision by chair holder), while any entry or promotion between 2010 and 2014 happened according to the TTS. In reality, some promotions took place after 2010 that did not follow the TTS, but these are considered to be a small, negligible minority. Data analysis consisted of three phases: descriptive analyses, testing for differences before and after the introduction of TTS, and extrapolations into the future.

### Descriptive analyses

For each time step (e.g. 2009 to 2010, 2010 to 2011, etc.), female and male entries were counted to investigate chances of women and men being appointed at an academic position higher than PhD student or junior researcher. Next, promotions at each rank level were counted for male and female staff. Entry and promotion rates were calculated as the absolute number divided by the total number of (female or male) employees in the source from which the promotions or appointments took place (e.g., the female promotion rate from UD2 to UD1 in the period 2010 to 2011 was measured as the total amount of female promotions in that period divided by the amount of female UD2s in 2010).

### Statistical tests

For testing the hypothesis, we adopted a natural experiment approach. A natural experimental design is typically applied to study relationships between real-world phenomena that are impossible to study in laboratory experiments [35]. If two conditions (i.e., presence and absence of tenure track system) exist in the same context (i.e., the same university), and the effects (i.e., ratio of promotion rates women/men to higher positions) can be adequately assessed, an experimental design can still be a useful approach. In our experimental design, the control condition is the situation before tenure track implementation. The intervention is the implementation of the tenure track system. Hence, the experimental condition is the situation after tenure track condition. The effect is the ratio of promotion rates of women divided by the promotion rates of men. The internal validity of natural experiments does not necessarily equal the internal validity of laboratory experiments (as the researchers have less opportunities to rule out potential confounds). Technically, for a natural experiment, researchers need to assume that other factors do not systematically covary between the two conditions. The control and intervention conditions occur at the same institution, and are applied to the same kind of individuals. Yet, the time frame of the two conditions is different, and hence some covariates (e.g. change in opinion or affirmative action) might occur (see discussion for elaboration). On the other hand, the ecological validity of natural experiments is superior relative to laboratory experiments, since actual, real-world phenomena are studied and hence the problem of whether relationships observed in laboratories exist in the much more complex world “out-there” does not exist.

We constructed a dataset in which the units of analysis were promotion chances. Theoretically, each individual in a rank lower than professor (PH) has a chance to be promoted to a higher position in each year. Yet, practically and habitually, tenure track staff applies for promotion after a period of three years in a specific rank. Therefore, the number of promotion chances is set at one third of the number of individuals in a specific position. The promotion chances have three attributes: occurring before or after TTS introduction, actualized or not, and for a female or for a male. We used chi-square to test for differences in promotion chances between women and men, both before and after TTS introduction, and within women and men before and after TTS introduction, as to determine whether promotion ratios women/men have changed. We used φ as the associated effect size measure.

### Extrapolation

**Fig 1:**
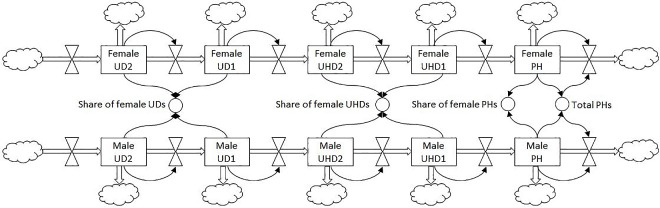
Stock and flow diagram.

Finally, extrapolations into the future (2025) were performed according to several scenarios with varying assumptions about promotion and appointment rates, to see if and when equal shares of male and female UDs, UHDs and PHs can be reached due to TTS policy as a standalone measure. Because the system exhibits a positive feedback (the larger the source in the previous level, the larger the flow to the next level), a linear extrapolation from past trends is not sufficient. Instead, we used a system dynamics approach, which allows exploring the development of a system over time by describing it in terms of stocks and flows. The stocks are, in this case, the amount of female and male scientific employees in the various ranks, while the flows represent the promotions of female and male staff from one rank to a higher one. The conceptual model is shown in Fig 1, and is subject to the following simplifications: (a) we did not take into account additional inflow from outside the university into ranks UD1 to PH, which is in line with the university’s TTS policy; and (b) we assumed that the total outflow equals the total inflow, and that outflow is distributed over all stocks proportional to the size of the stocks. We did not assume that women quit more often than men (as supported by findings of [36]). The flows were derived from the empirical observations of the period 2010–2014, and the initial stocks were those of 2014. Shares of female UDs, UHDs and PHs are output variables.

### Ethics statement

The Human Resource Management department of Wageningen University was responsible for data compilation, and database management and construction. Data was anonymized and de-identified prior to access and analysis by the authors.

## Results

Staff being assigned as UD2 (from 2010: “Staff entering the tenure track”), differentiated per year and sex (Table 1). The pool of potential internal candidates for UD2 positions at Wageningen University (i.e., PhD students and junior researchers) has grown over the years, and in particular the pool of female candidates. In 2009, the female group of PhD students and junior researchers employed at Wageningen University was, for the first time in history, larger than the group of male PhD students and junior researchers. Yet, both before and after TTS introduction, more males (44 before, 53 after TTS) were selected for UD2 positions than were females (33 before, 34 after).

**Table 1:** UD2 appointments before and after introduction of the TTS.

	Before introduction TTS				After introduction TTS			
	2006–2007	2007–2008	2008–2009	2009–2010	2010–2011	2011–2012	2012–2013	2013–2014
Female staff								
Pre-UD in prev. year	341	378	419	488	494	565	606	600
To UD2	5	12	7	9	15	7	3	9
Rate entering	1.5%	3.2%	1.7%	1.8%	3.0%	1.2%	0.5%	1.5%
Average over period				**2.0%**				**1.6%**
Male staff								
Pre-UD in prev. year	437	437	447	481	487	509	536	514
To UD2	10	12	11	11	14	13	12	14
Rate entering	2.3%	2.7%	2.5%	2.3%	2.9%	2.6%	2.2%	2.7%
Average over period				**2.4%**				**2.6%**

Tables 2 and 3 show the development of female and male staff from UD2 to higher ranks. The figures suggest an increase in promotion rates after TTS introduction for both women and men. Due to the small number of promotions, particularly within the female sample, the time series are highly volatile and differences between male or female promotions before and after introduction of TTS can only be tested at an aggregate level.

**Table 2:** Female promotions before and after introduction of the TTS.

	Before introduction TTS				After introduction TTS			
	2006–2007	2007–2008	2008–2009	2009–2010	2010–2011	2011–2012	2012–2013	2013–2014
UD2 in previous year	24	25	33	36	36	46	41	30
UD2 to UD1	2	0	3	2	2	10	9	7
Rate UD2 to UD1	8.3%	0.0%	9.1%	5.6%	5.6%	21.7%	22.0%	23.3%
Average over period				**5.9%**				**18.3%**
UD1 in previous year	52	56	58	57	52	50	59	63
UD1 to UHD2	1	0	0	5	3	1	4	7
Rate UD1 to UHD2	1.9%	0.0%	0.0%	8.8%	5.8%	2.0%	6.8%	11.1%
Average over period				**2.7%**				**6.7%**
UHD2 in previous year	3	4	5	6	12	9	10	16
UHD2 to UHD1	0	0	0	0	3	1	2	1
Rate UHD2 to UHD1	0.0%	0.0%	0.0%	0.0%	25.0%	11.1%	20.0%	6.3%
Average over period				**0.0%**				**14.9%**
UHD1 in previous year	16	16	12	12	11	16	17	19
UHD1 to PH2	0	0	0	1	0	0	0	0
Rate UHD1 to PH2	0.0%	0.0%	0.0%	8.3%	0.0%	0.0%	0.0%	0.0%
Average over period				**1.8%**				**0.0%**

**Table 3:** Male promotions before and after introduction of the TTS.

	Before introduction TTS				After introduction TTS			
	2006–2007	2007–2008	2008–2009	2009–2010	2010–2011	2011–2012	2012–2013	2013–2014
UD2 in previous year	22	26	33	40	43	50	53	52
UD2 to UD1	3	3	2	2	5	6	11	14
Rate UD2 to UD1	13.6%	11.5%	6.1%	5.0%	11.6%	12.0%	20.8%	26.9%
Average over period				**8.3%**				**18.2%**
UD1 in previous year	177	182	180	173	160	153	153	149
UD1 to UHD2	2	6	6	6	8	4	7	8
Rate UD1 to UHD2	1.1%	3.3%	3.3%	3.5%	5.0%	2.6%	4.6%	5.4%
Average over period				**2.8%**				**4.4%**
UHD2 in previous year	13	16	22	28	30	33	35	40
UHD2 to UHD1	0	0	0	2	6	4	2	6
Rate UHD2 to UHD1	0.0%	0.0%	0.0%	7.1%	20.0%	12.1%	5.7%	15.0%
Average over period				**2.5%**				**13.0%**
UHD1 in previous year	111	107	97	96	95	93	95	90
UHD1 to PH2	3	3	0	1	0	0	1	1
Rate UHD1 to PH2	2.7%	2.8%	0.0%	1.0%	0.0%	0.0%	1.1%	1.1%
Average over period				**1.7%**				**0.5%**

Before TTS introduction, the ratio of promotion rates women/men was 1.14. Within this ratio, the promotion rates of females (14 out of 138 chances) and males (39 out of 441) (Table 4) were statistically equal (*χ*^2^ = .21, *p* = .64, φ = .02), hence, the ratio is not statically different from 1. After TTS introduction, the ratio of promotion rates was 1.64, and the promotion rate of females was larger than the promotion rate of males (*χ*^2^ = 9.99, *p* = .002, φ = .13), indicating that the ratio is statistically larger than 1. Hence, by extension, the ratios before and after TTS are different. For both females (*χ*^2^ = 19.06, *p* < .001, φ = .25) and males (*χ*^2^ = 18.41, *p* < .001, φ = .14) promotion rates have increased after the introduction of TTS, the effect size for females being larger than the effect size for males. The effect sizes are small to almost medium [37]. Absence of large effect sizes is a logical consequence of the subject matter: As promotion rates in general are small (also in TTS, which is probably designed such), effect sizes are likely to be small too. Only if a relatively large portion of promotion chances are actualized, larger effect sizes can be expected.

**Table 4:** Promotion rates females and males before and after TTS introduction.

	Gender	# of promotion chances	# of promotions	# of non-promotions	Promotion rates^1^
Before TTS	Female	138	14	124	10.1%^a^
	Male	441	39	402	8.8%^a^
After TTS	Female	162	50	112	30.9%^b^
	Male	441	83	318	18.8%^c^

^1^ Different superscripts indicate statistically significantly different rates.

**Fig 2:**
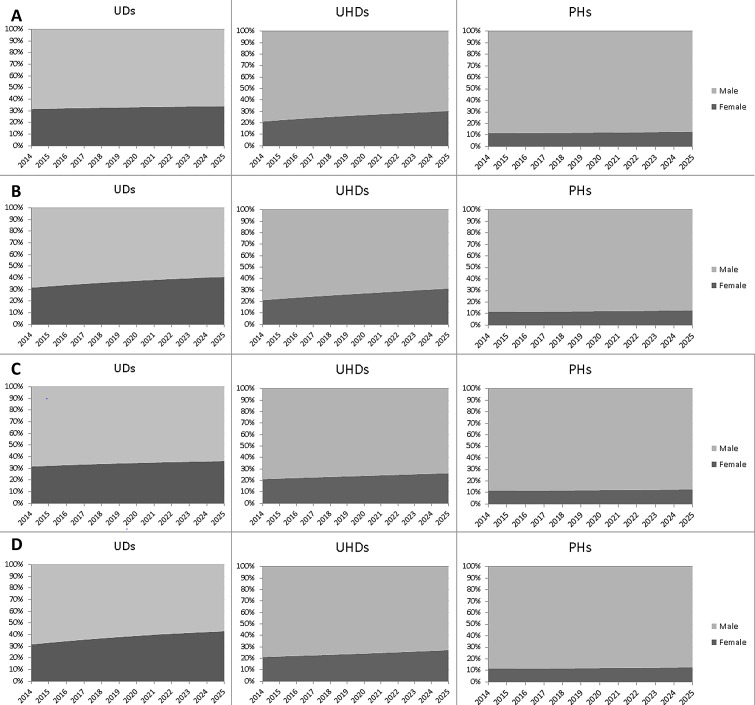
Scenarios of female shares of UD, UHD and PH.

Fig 2 displays the development of male and female shares for the (lumped) categories UD, UHD and PH until 2025 for various scenarios of entry and promotion rates (as specified in Table 5). In scenario A, entry and promotion rates as observed in the period 2010–2014 have been extrapolated to the year 2025. Hereby, the promotion rate of women from UHD1 to PH has been set to 0.5% (the male rate), to avoid having no entries at all at PH level for women. In scenario B, the entry rates of men and women are set to an average value of 11 per year, while the promotion rates are assumed to be as observed for the period 2010–2014. In scenarios C and D, the slight advantage of women in the TTS is no longer present (simulating a fade out of what could be a catch-up effect: see discussion), and promotion rates for women are set to being the same as those of men.

**Table 5:** Entry and promotion rates used as input for four different future scenarios.

Scenario	Entry rate		UD2 to UD1		UD1 to UHD2		UHD2 to UHD1		UHD1 to PH	
	Male	Female	Male	Female	Male	Female	Male	Female	Male	Female
A	13.3	8.5	17.8	18.1	4.4	6.4	13.2	15.6	0.5	0.5
B	11.0	11.0	17.8	18.1	4.4	6.4	13.2	15.6	0.5	0.5
C	13.3	8.5	17.8	17.8	4.4	4.4	13.2	13.2	0.5	0.5
D	11.0	11.0	17.8	17.8	4.4	4.4	13.2	13.2	0.5	0.5

Numbers in this table are collected from Tables 1, 2 and 3, rightmost columns.

Fig 2 shows that in all four scenarios the share of female PHs remains virtually unchanged. In the scenario that is most favourable towards equal representation of women (scenario B), 12.9% of all PHs is female in 2025, relative to 11.6% in 2014. The share of female PHs is fairly insensitive to the different scenarios: the worst-case scenario (Scenario C) shows an increase in the female PH share to 12.7%. The female UHD share shows more variability between the scenarios: under Scenario B, the UHD population in 2025 will consist for 31.4% of women (compared to 21.2% in 2014), while under Scenario C it would amount to 26.3%. The entry rates affect the distribution of the UD population to a considerable extent, but this effect becomes smaller at the UHD level (1 percentage point in 2025) and is negligible at the PH level.

## Conclusion and Discussion

Overall, a positive impact of the TTS on female promotion rates was observed. TTS increases the promotion rates of women as well as men, but more so of women, as suggested by the effect sizes (φ = .25 for females versus φ = .14 for males) and hence there is a difference in ratios of promotion rates of women/men before (1.14) and after (1.64) TTS introduction. These findings confirm our hypothesis. Yet, we expected that the increase in women’s promotion rate would be due to cancelling-out of gender bias disfavouring women before TTS introduction, and hence women’s and men’s rates would become (more) equal. However, before the TTS was introduced, promotion rates for female staff were statistically equal to those of their male colleagues, while since the introduction of the TTS promotion rates of women have been *higher* than those of men. Thus, instead of disparity disfavouring women before TTS (expected), there is disparity disfavouring men after TTS (observed).

Various explanations for higher promotion rates of women compared to men after TTS introduction are conceivable. First, close inspection of data indicates that the relative share amongst males in UHD1 positions is much larger than the equivalent share amongst females (Tables 2 and 3)–a figure that in itself suggests strong gender bias. As the requirements for being promoted from UHD1 to PH are high, these promotions are rare and many of these males might have arrived at their top level already. This could suppress the aggregate promotion rate of men, relative to that of women. Additional analysis indeed suggests the promotion rates ratio decreased from 1.64 to 1.38 after TTS introduction if the UHD1 level is excluded. Still, the difference between women and men is statistically significant (*χ*^2^ = 4.69, *p* = .03, φ = .10), and hence this explanation provides a partial account only. Second, it might be a catch-up effect of female employees who had been assigned a too low rank previously. Such a catch-up effect is expected to level off after a couple of years, and hence inspection of future figures of Wageningen University can shed light on this explanation. Third, it might be an effect of affirmative action measures, such as premiums awarded by the Dutch Science Council for excellent female scientists, provided they are appointed to UHD (responsible for the accelerated promotion of at least four female scientists at Wageningen University during the TTS period). The affirmative action effect depends on gender policies of the university and external funding agencies, and hence future variation in these would then lead to changes in promotion rates. Fourth, higher promotion rates for women after TTS introduction might simply be a consequence of a higher relative share of women deserving promotion, compared to the share of men. The total number of female staff at Wageningen University is only about one third of the total number of male staff. Research suggests that capacities for academic work are not different between men and women [38], that parenthood does not have a detrimental effect on women’s academic careers relative to men’s careers [10,39,40], and that girls overall outperform boys at all education levels [41]. The small share of women at Wageningen University therefore suggests that their selection has probably been more rigorous in the past than selection of men (due to women having to compensate for bias favouring men, in order to be hired). Consequently, capacity and performance of women at Wageningen University might, on average, be slightly higher than those of men, and hence women’s promotion rate becomes higher in the TTS. Future research could include performance data into the analyses to check the merit of this explanation.

In spite of the higher promotion rates for women in the TTS, the scenarios reveal that rates are insufficiently large to ensure a balanced representation of women scientists in senior academic positions within a reasonable timeframe. The extrapolations (Fig 2) show that it will take a considerable time before equal representation of women is achieved by natural turnover. Striking is that the differences between best (Scenario B) and worst case (Scenario C) are not that large, especially for the trends in PHs. Since promotion rates are generally small, near-future developments remain to be dominated by the current distribution of male and female staff (approximately 3 to 1). Moreover, as appointment of new UD2s suggest gender bias also after TTS entry (34 women versus 53 men newly appointed), it is unlikely that eliminating gender bias in promotions as a standalone measure would lead to a drastically increased representation of women in higher positions at Wageningen University. The procedure for appointing new staff is different than the procedure for promotion. Staff can pursue promotion irrespective of their chair holder’s approval to do so. Candidates for new appointments, on the other hand, always need to be nominated by a chair holder. Hence, system 1 influences, with higher changes of stereotyped gender bias, might play a larger role here.

In spite of the slowness of the process, the presented numbers give rise to some optimism with respect to long term prospects for reaching the ideal of gender equality. However, some critical remarks are in place as well. First, it should be noted that the presented results do not prove that for female employees of Wageningen University it is as easy to meet tenure track criteria as it is for male employees. Given the literature suggesting that women (a) have a higher chance of paper rejections in single-blind reviewed journals [42], (b) are generally less positively evaluated by students [16], and (c) are assessed as being less senior and qualified in grant application reviews [15], female scientists may still have more difficulties in meeting the tenure track criteria than male colleagues with equal qualities.

The influences of system 1 and system 2 thinking on decision making on promotions are not directly tested by the current research, as we did not tap into thinking processes. The conceptual distinction served as a background explanation of how gender bias might exist in promotion appointments, and why it could decrease in a TTS. As promotion rates before TTS were not significantly different for women and men, the figures do not directly suggest system 1 generated stereotyped gender cognitions influencing decisions. Yet, if our argument that unequal distribution of women and men implies more rigorous selection of women, and on average a slightly higher capacity of women (or at least a relatively larger portion of high-capacity individuals amongst women), is sound, the equal promotion rates might still reflect gender bias due to a larger influence of system 1 before TTS. Also, following the same argument, higher promotion rates after TTS might indeed be explained by increasing the role of system 2 thinking and thus decreasing gender bias.

The psychological theory of two systems of thinking is useful for understanding and studying gender issues in academia and beyond. If offers an understanding of how individuals (both women and men) have non-deliberately internalised gender bias, and why it can continue to exist, also in individuals who explicitly adhere to the ideal of gender equality. When we think of ourselves, we think of system 2, that is, we identify with system 2, and the working of system 1 is by and large not open to introspection [27]. Moreover, the theory fosters comprehending how cultural stereotypes and the associated biases can become internalized, and it offers a framework to think about effective solutions. For instance, the theory can also explain why rigorous double blind review increases representation of female authors [42]. Our theoretical perspective pertains to the psychological level of analysis. The cultural level (e.g., how subtle social rules constituting gender differences are produced, propagated, transformed, and maintained by institutions and other forms of power) is equally important [43], and is complementary. As an example, clarity of evaluation is deemed an organizational factor that supports the advancement of women in science, a claim that is perfectly in line with our findings [28]. The theory of system 1 and 2 provides an entry for connecting these two levels of analysis, by offering an explanation of how the cultural framing of gender can influence psychological decision-making. Finally, positive consequences of attempts to decrease system 1 influences in decision-making relevant to academic careers extend beyond gender equality, to include for instance avoiding race bias as well.

The database that was made available by the HRM department did not allow us to differentiate between different scientific domains. Such differentiation could affect our results whenever some domains show structurally deviating promotion rates as well as structurally deviating female staff shares (without the two being directly causally related). From an informal conversation with our rector, we have understood that the Environmental and Social Science departments show slightly elevated promotion rates, as compared to the other departments (Arthur Mol, personal communication). However, these two groups together have a share of female employees that is similar to the overall share at Wageningen University, and so we consider it unlikely that our results would have been different if we would have been able to differentiate the analysis per scientific domain.

As our data are confined to a single university, findings cannot blindly be generalized to other contexts. Gender distributions of staff, ratios of promotion rates between the sexes, and gender representations amongst those taking promotion decisions might be very different at other institutions, and all of these differences might lead to different figures. Still, we believe that the present findings have wider implications relevant to other academic institutions, irrespective of differences in specific figures. It is likely that TTS leads to an increase in promotion rates of women to higher scientific positions, which can probably be ascribed to the more objective assessment, and less system 1 generated bias, compared to a traditional system in which promotions were based on the chair holders’ assessment. Nevertheless, in institutions where underrepresentation exists, equal gender distributions in higher academic positions will not occur due to equal promotion rates or even rates favouring women only. The promotion rates are such that natural turnover only very slowly improves the gender balance in higher scientific positions. Additional measures, such as equal entry rates and additional appointments of women directly at higher ranks, are needed as well.
